# Circulating CD56^bright^ NK cells inversely correlate with survival of melanoma patients

**DOI:** 10.1038/s41598-019-40933-8

**Published:** 2019-03-14

**Authors:** Kaat de Jonge, Anna Ebering, Sina Nassiri, Hélène Maby-El Hajjami, Hajer Ouertatani-Sakouhi, Petra Baumgaertner, Daniel E. Speiser

**Affiliations:** 1 Department of Fundamental Oncology, University of Lausanne, Epalinges, Switzerland; 2 Department of Oncology, University Hospital Center (CHUV), Lausanne, Switzerland; 3 Swiss Institute of Bioinformatics (SIB), Bâtiment Génopode, Lausanne, Switzerland

## Abstract

The roles of NK cells in human melanoma remain only partially understood. We characterized NK cells from peripheral blood *ex vivo* by flow cytometry obtained from late stage (III/IV) melanoma patients. Interestingly, we found that the abundance of CD56^bright^ NK cells negatively correlate with overall patient survival, together with distant metastases, in a multivariate cox regression analysis. The patients’ CD56^bright^ NK cells showed upregulation of CD11a, CD38 and CD95 as compared to healthy controls, pointing to an activated phenotype as well as a possible immune regulatory role in melanoma patients. After stimulation *in vitro*, CD56^bright^ NK cells produced less TNFα and GMCSF in patients than controls. Furthermore, IFNγ production by the CD56^bright^ NK cells correlated inversely with overall survival. Our results highlight that abundance and function of CD56^bright^ NK cells are associated with melanoma patient survival, emphasizing the potential of NK cell subsets for biomarker discovery and future therapeutic targeting.

## Introduction

Melanoma is, next to squamous cell carcinoma and basal cell carcinoma, one of the three major types of skin cancer^1^. Due to its highly metastatic potential, with metastases developing amongst others in lymph nodes, liver, lung and brain, the mortality rate is highest for melanoma amongst the three skin cancer types, although being the least frequent. While the number of people suffering from melanoma grows worldwide, the survival rates of metastatic melanoma patients remain inadequate. Treatments with immune checkpoint blocking antibodies such as Ipilimumab (anti-CTLA-4) and Nivolumab (anti-PD1) result in progressively increased rates of clinical responses. However, many patients still do not respond to available therapies^2^. Mechanisms of response to checkpoint blockade are not completely understood. Among other players, NK cells may potentially modulate immunotherapy effects^3^.

NK cells are part of the innate immunity branch of the group of innate lymphoid cells (ILC)^4^. They are potent killers of virally infected as well as cancer cells without needing prior sensitizations^5^. Moreover, they are potent cytokine producers^6^. In humans, NK cells can be divided into two main subsets, comprising the immature, poorly cytotoxic but cytokine-producing CD56^bright^, and the mature, cytolytic, weakly cytokine-producing CD56^dim^CD16^+^ NK cells. CD56^bright^ NK cells only make up around 10% of NK cells in the periphery; they are, however, the major subtype in tissues and second lymphoid organs^7^.

NK cells are often seen as a positive factor in the anti-tumour immune response since their involvement in immune surveillance has been shown multiple times^8–11^. For example, in rag mice that lack an adaptive immune system, the production of IFNγ by NK cells was found indispensable in the immune editing process^12^. Additionally, mice without natural killer cells are less able to reject several tumour cell lines, including B16 melanoma cells^8^. Moreover, injection of pre-activated murine NK cells persisted within the tumour and they were able, in combination with radiotherapy, to significantly reduce the growth of primary tumours and metastases^13^. In melanoma patients, NK cells have been studied in both the peripheral blood as well as in tissues, however they are yet insufficiently characterized and contradictory results have been found^14^. Some investigators have reported unaltered frequencies of CD56^bright^ and CD56^dim^ subsets in blood of metastatic melanoma patients^15,16^, whereas others have found a decrease in both subsets^17^. Moreover, peripheral NK cells appeared to have impaired IFNγ production and degranulation^15,18^. CD56^dim^ NK cells derived from blood expressed lower levels of the activation receptor NKG2D, and CD56^bright^ NK cells expressed higher levels of the inhibitory KIR receptor CD158b^15^. Fregni *et al*. reported decreased expression of the natural cytotoxicity receptor NKp46, in line with previous publications. However, they reported no significant differences between patient and healthy controls in the production of IFNγ and the degranulation marker CD107a^16^. NK cells are able to infiltrate primary tumours especially in the peritumoral area^16^. Tirosh *et al*. characterized the tumour micro-environment (TME) in metastatic melanoma by single cell analysis and found NK cells present in small numbers within the tumor^19^. The presence of total NK cells in the TME was found to positively correlate with clinical outcome in patients with colorectal carcinoma, gastric carcinoma and non-squamous lung carcinoma^20–22^. Little information is available about NK cell phenotype and functionality in primary melanoma. One study showed that NK cells within tumour-infiltrating lymphocytes (TILs) are CD56^bright^CD16^dim^, a phenotype that is also found in regulatory decidual NK cells^14^. Other studies have focused on the NK cell phenotype in tumour-infiltrated lymph nodes, reporting an enrichment of CD57^+^KIR^+^CD56^dim^ NK cells, which are very efficient at killing autologous melanoma cell lines^17^, suggesting a fully mature and effector phenotype. The ratio of CD56^dim^CD57^+^ to CD56^bright^CD57^+^ seems to be biologically important and associated with survival in stage III melanoma patients^17^. Messaoudene *et al*. reported the presence of activated NK cells in tumour-infiltrated lymph nodes, expressing higher levels of NKp46, NKG2D, NKp44, DNAM-1 and NKp30. Moreover, they found a population of CD56^bright^ NK cells expressing CD16 as well as other activation receptors and KIRs. This population could also be found in lymph nodes adjacent to tumour-infiltrated lymph nodes but not in the blood^23^.

NK cell based therapies have not been successful in solid tumors^24^. However, NK cells are associated with durable responses after various therapies. NK cells are mediators of antibody-dependent cell cytotoxicity and have been shown to interact with α-CTLA-4 antibody, whilst at the same time inducing NK cell maturation^25^. A new experimental therapy targeting both CD8 T cells and NK cells by blocking the NKG2A receptor has shown early promising results in squamous cell carcinoma patients^26^. Interestingly, the frequency at baseline of CD56^bright^ NK cells in blood of melanoma patients treated with Ipilimumab was negatively correlated with overall survival^27^.

Our goal was to characterize circulating NK cells and their subpopulations as well as their functionality in late stage (III/IV) melanoma patients. We hypothesized that apart from a reduced anti-tumour function they could also play regulatory roles. We found that CD56^bright^ NK cells are negatively correlated with overall survival of the melanoma patients. Therefore, we determined the phenotypic and functional characteristics of circulating CD56^bright^ NK cells, improving the basis for a better understanding of NK cell tumour biology and future optimization of cancer immunotherapy.

## Results

### The frequency of circulating CD56^bright^ NK cells correlates inversely with patient survival

**Figure 1:**
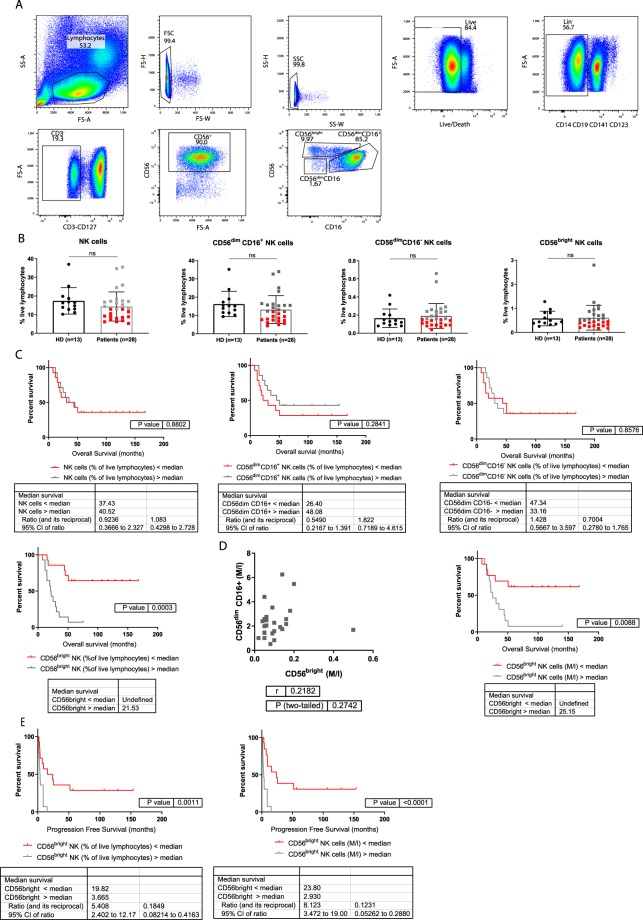
Frequencies of NK cells in melanoma patients and healthy controls. (**A**) Representative dot plots of the gating strategy used. Lymphocytes were selected using forward (FSC) and side scatter (SSC), afterwards doublets were gated out and live cells were selected. A series of negative selections was performed, first gating out DCs, monocytes and B cells using a lineage cocktail, next T cells and ILCs were gated out using CD3 and CD127. Total NK cells were positively selected using CD56 (total NK cells), this population can be further divided into CD56^bright^, CD56^dim^CD16^+^ and CD56^dim^CD16^−^ NK cells. (**B**) Histograms of the frequencies of total NK cells, CD56^bright^, CD56^dim^CD16^+^ and CD56^dim^CD16^−^ NK cells, as measured by flow cytometry in PBMC samples of 28 melanoma patients and 13 healthy donors. Frequencies of the patients with values lower than the median are indicated in red, and those higher than the median are indicated in grey. (**C**) Kaplan-Meier curves of overall survival, of patients with high (grey) vs. low (red) percentages of total NK cells, CD56^dim^CD16 + , CD56^dim^CD16^−^ and CD56^bright^ NK cells, with the median as cut-off. (**D**) Absolute numbers of corresponding CD56^dim^CD16^+^ and CD56^bright^ NK cells represented in a xy-plot. Kaplan-Meier curves of overall survival with high (grey) vs. low (red) numbers of CD56^bright^ NK cells, with the median as cut-off. E. Kaplan-Meier curves of progression free survival with high (grey) and low (red) frequencies and absolute numbers of CD56^bright^ NK cells with the median as cut-off. ns not significant, * p < 0.05, ** p < 0.01, *** p < 0.001, **** p < 0.0001.

We studied 29 late stage (III/IV) melanoma patients^28^ included in a vaccine clinical trial, and focused on NK cells before treatment start. PBMCs were analysed directly *ex vivo* by flow cytometry, using the gating strategy as represented in Figure 1A. 1 patient was excluded due to technical issues. 13 healthy donors were included as controls. We found no difference in the frequency of total NK cells as well as CD56^bright^, CD56^dim^CD16^+^ and CD56^dim^CD16^−^ NK cells between patients and healthy donors (Fig. 1B). Interestingly, patients with high frequencies or absolute numbers of CD56^bright^ NK cells had significantly shorter overall survival than patients with low frequencies or absolute numbers (Fig. 1C,D). We did not find a significant correlation between absolute numbers of CD56^bright^ and CD56^dim^CD16^+^ NK cells, indicating that the negative correlation between overall survival and the number of CD56^bright^ NK cells is not a result of corresponding low numbers of CD56^dim^CD16^+^ NK cells (Fig. 1D). Frequencies and numbers of peripheral CD56^bright^ NK cells did not only inversely correlate with overall but also progression free survival (Fig. 1E). No significant correlation was observed between patient survival and total NK cells, or CD56^dim^CD16^+^ or CD56^dim^CD16^−^ NK cells (Fig. 1C). Frequencies of NK cells and their subsets were similar in healthy donors and melanoma patients at stage III and IV (Suppl. Fig. 1A). Numbers of CD56^bright^ NK cells does not significantly differ between stage III and IV patients (Suppl. Fig. 1B). Frequencies of CD56^bright^ NK cells are not significantly different between patients having received any previous treatment (chemo, radio or immunotherapy) (Suppl. Fig. 1C–E). Since CD56^bright^ NK cells seem to be a prognostic factor for survival, we decided to characterize them in more detail.

### CD56^bright^ NK cells have an activated phenotype in patients

**Figure 2:**
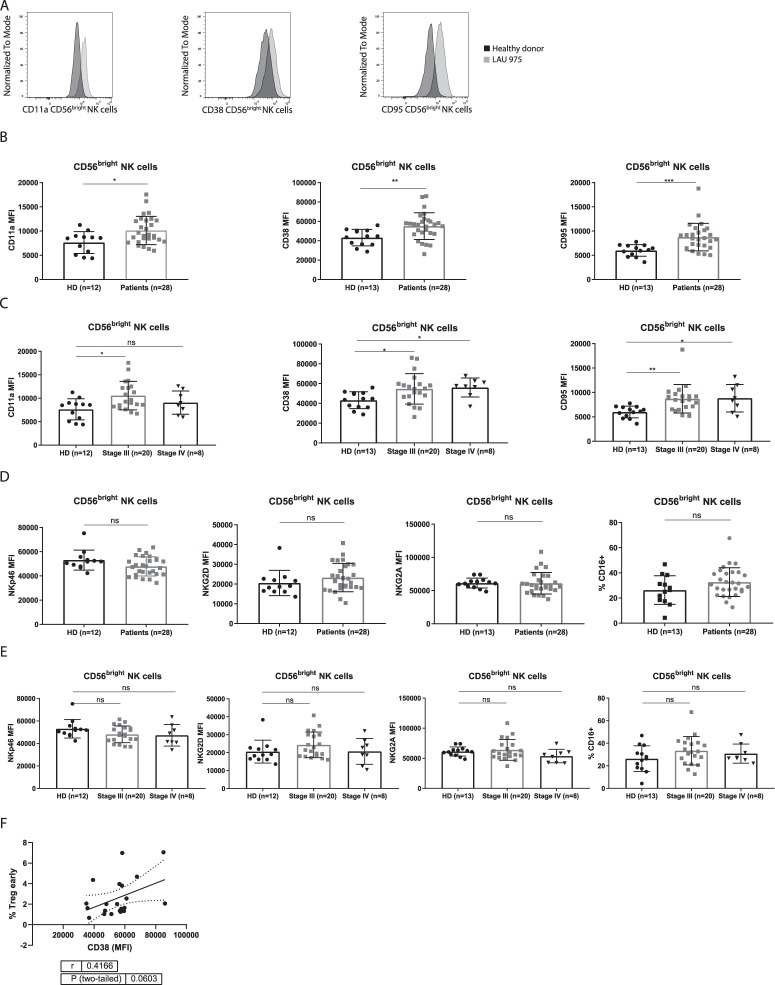
Phenotypic characterization of CD56^bright^ NK cells by flow cytometry. (**A**) Representative histograms of the expression level (Median Fluorescent Intensity, MFI) of CD11a, CD38 and CD95 on CD56^bright^ NK cells. The histograms show overlays; patient LAU975 (grey) and healthy donor 7 (black). (**B**) Summary histograms of the expression levels (MFI) of CD11a, CD38 and CD95. (**C**) Distribution of the expression levels (MFI) of CD11a, CD38 and CD95 from patients with late stage (III/IV) melanoma and healthy donors. (**D**) Summary histograms of the expression levels (MFI) of NKp46, NKG2D and NKG2A. (**E**) Distribution of the expression levels (MFI) of NKp46, NKG2D and NKG2A from patients with late stage (IIII/V) melanoma and healthy donors. (**F)** Frequencies of regulatory T cells (CD25^hi^FoxP3^+^CD127^−^) in the blood of melanoma patients before vaccination (n = 21) correlating with the expression levels of CD38 (MFI) on CD56^bright^ NK cells (n = 21) from the same patients. ns not significant, *p < 0.05, **p < 0.01, ***p < 0.001, ****p < 0.0001.

Patient and healthy control NK cells were analysed for the expression levels of multiple NK cells markers, inhibitory and activating receptors as well as activation markers by flow cytometry. As compared to healthy donors, circulating CD56^bright^ NK cells of melanoma patients showed elevated expression of CD11a, CD38 and CD95, as measured directly *ex vivo* (Fig. 2A,B). The observations were consistent after patients were stratified according to their disease status: stage III or IV (Fig. 2C). We did not observe any difference in the expression levels of NKG2A, NKp46 or NKG2D (Fig. 2D), and these markers were also consistently expressed in patients at different disease stages (Fig. 2E). We did not observe expression of KLRG1, CD158b1,b2,j (a pan KIR marker) or CD57 (data not shown). Elevated expression of CD11a, CD38 and CD95 indicates an activated phenotype. Moreover, CD38 is part of the adenosine pathway^29^; adenosine is a immunoregulatory factor that promotes regulatory T cells and inhibits conventional T cell function^30^. We found a trend for increased CD38 expression on CD56^bright^ NK cells and the prevalence of regulatory T cells (Fig. 2F). Despite the elevated expression of CD95 (FasR) we did not see evidence for increased apoptosis of the CD56^bright^ NK cells as measured by Annexin V (Suppl. Fig. 2A). CD56^dim^CD16^+^ NK cells also seem to have an activated phenotype as characterized by higher expression of CD11a, CD95 and NKG2D. They are less highly differentiated than their counterpart in healthy donors as characterized by a lower frequency of CD57 expressing cells (Suppl. Fig. 2B,C). Levels of NKp46, NKG2A, KLRG1, CD38, CD95 and CD158b1,b2,j on CD56^dim^CD16^+^ NK cells were not found to be different between patients and healthy donors (Suppl. Fig. 2B). Finally, we found no expression of PD-1 and CTLA-4 by either CD56^bright^ or CD56^dim^CD16^+^ NK cells (data not shown).

### Pro-inflammatory cytokine and chemokine production is partially affected in CD56^bright^ NK cells

**Figure 3:**
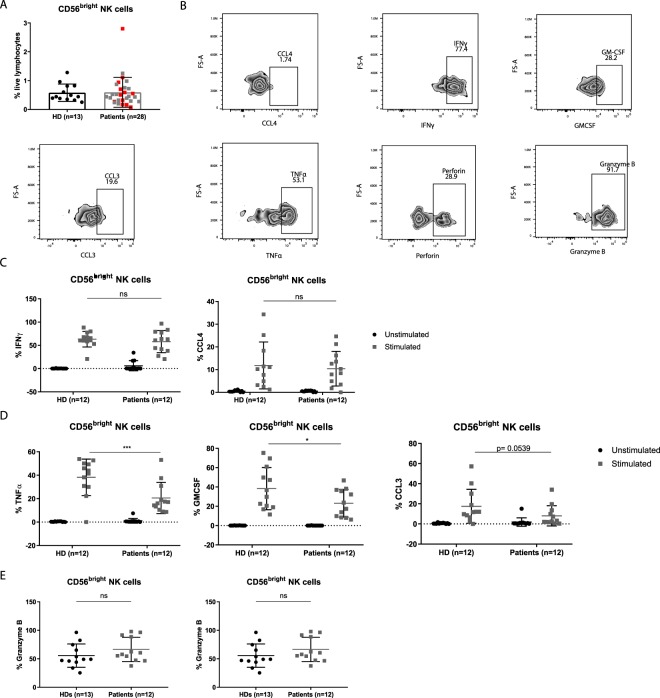
Functional characterization of CD56^bright^ NK cells after stimulation (4 hours) with PMA/Ionomycin. (**A**) Histogram of the frequencies of CD56^bright^ NK cells in patients and healthy donors. Patients included in subsequent functional experiments are indicated in red. (**B**) Representative dot plots of CCL4, IFNγ, GMCSF, CCL3 and TNFα after 4 hours of PMA/Ionomycin stimulation, and of perforin and granzyme B without stimulation of patient LAU627. (**C**) Histograms of IFNγ and CCL4 production (%) (CD56^bright^ NK cells) in healthy donors and patients before and after stimulation. (**D**) Histograms of TNFα, GMCSF and CCL3 production (%) (CD56^bright^ NK cells) in healthy donors and patients before and after stimulation. (**E**) Histograms of granzyme B and perforin (%) (CD56^bright^ NK cells) in healthy donors and patients before stimulation. ns not significant *p < 0.05, **p < 0.01, ***p < 0.001, ****p < 0.0001.

Since CD56^bright^ NK cells have an activated phenotype we wondered if they are also more capable of producing cytokines and chemokines than NK cells from healthy donors. Previous publications have shown that NK cells are able to produce an array of cytokines, including Th1, Th2 as well as regulatory cytokines^31^. We used samples from 12 patients representing the whole patient population based on their frequencies of CD56^bright^ NK cells (Fig. 3A), and the same previously used 13 healthy donors as controls. We stimulated NK cells for 4 hours with PMA/Ionomycin and analysed them by flow cytometry. ILCs were excluded by gating out CD127-positive cells. Previous studies have reported decreased functionality of peripheral blood NK cells in melanoma patients^15^. We did not detect any differences between the amounts of IFNγ or CCL4 produced by CD56^bright^ NK cells from patients and healthy donors (Fig. 3B,C). In contrast, we found lower production of TNFα and GMCSF (and a trend for CCL3) in patients as compared to healthy donors (Fig. 3B,D). We did not find differences for granzyme B and perforin expression before stimulation, suggesting similar cytolytic capacity (Fig. 3B,E). IFNγ, GMCSF, perforin, granzyme B, CCL4 and NKp44 production by CD56^dim^CD16^+^ cells did not differ between patients and healthy donors. In turn, the patients produced lower levels of TNFα and CCL3 (Suppl. Fig. 3A,B). Finally, we did not observe any production of IL-10, IL-2, LTα, IL-4, IL-13, IL-22 and IL-5 in either subset (CD56^bright^ and CD56^dim^CD16^+^) (data not shown). Even though CD56^bright^ NK cells display an activated phenotype based on surface markers, they are not more functional than healthy controls, they are even impaired in the production of a number of cytokines.

### Multivariate analysis shows significant correlation of CD56^bright^ NK cells with overall patient survival

We used the clinical as well as phenotypical and functional data from our cohort to perform a univariate Cox regression analysis for overall patient survival, with the aim to identify significant clinical and immunological factors. One of the immunological parameters is the frequency of Melan-A specific CD8^+^ T cells at baseline, as previously described^28^. Interestingly, we found that presence of distant metastases, frequency of circulating CD56^bright^ NK cells, and production of IFNγ by CD56^bright^ NK cells are inversely associated with overall patient survival (Table 1). To test if these three parameters contribute individually to survival we performed a multivariate Cox regression analysis. Since we only had 11 paired samples for our functional analyses, we decided to not analyse the impact of IFNγ production by CD56^bright^ NK cells any further. Our multivariate analysis showed that both, the presence of distant metastases and the frequency of CD56^bright^ NK cells correlated significantly with survival (Table 1). A similar result was obtained using absolute numbers of CD56^bright^ NK cells instead of frequencies (data not shown). The frequency of CD56^bright^ NK cells was similar in patients with or without distant metastases (Suppl. Fig. 4). Together, our data suggest that the fraction of circulating CD56^bright^ NK cells may hold prognostic value in melanoma patients.

**Table 1:** Univariate and multivariate analysis of overall survival by the Cox proportional hazards method. *p < 0.05, **p < 0.01, ***p < 0.001, ****p < 0.0001.

	Univariate Cox analysis				Multivariate Cox analysis			
	beta	HR	95% CI for HR	P value	beta	HR	95% CI for HR	P value
Age (years)	−0.012	0.99	0.96–1	0.42				
LDH (mgEq/l)	−0.0025	1	0.99–1	0.59				
Sex	−0.17	0.84	0.33–2.2	0.73				
Lymph node metastases	−0.23	0.8	0.28–2.3	0.67				
Satellite metastases	0.84	2.3	0.9–6	0.082				
Distant metastases	1.1	3.1	0.97–6.5	0.031*	1.077	2.937	1.028–8.393	0.044*
N stage	0.83	2.3	0.66–7.9	0.19				
DTH	−0.95	0.39	0.13–1.1	0.082				
NED or ED at study entry	0.92	2.5	0.97–6.5	0.059				
Previous radiotherapy	0.096	1.1	0.32–3.8	0.88				
Previous chemotherapy	−0.26	0.77	0.22–2.7	0.68				
Previous immunotherapy	0.076	1.1	0.42–2.8	0.88				
Any previous therapy (Radio/Chemo/Immuno)	−0.016	0.98	0.39–2.5	0.97				
MelanA^+^ CD8 (% CD8^+^ T cells)	−0.61	0.54	0.12–2.6	0.44				
CD56^Bright^ NK cells (% live lymphocytes)	2.9	18	2.4–140	0.005**	2.963	19.364	2.293–153–524	0.007**
CD11a (MFI)	1.3	3.7	0.049–280	0.56				
NKp46 (MFI)	4.7	110	0.08–160 000	0.2				
NKG2D (MFI)	1.9	6.4	0.19–220	0.3				
NKG2A (MFI)	2.7	14	0.07–2900	0.33				
CD38 (MFI)	0.42	1.5	0.015–160	0.86				
CD95 (MFI)	0.96	2.6	0.027–260	0.68				
CD16 (% CD56^bright^ NK cells)	2.5	12	0.43–350	0.14				
Granzyme B (% CD56^bright^ NK)	4.7	110	0.54–20 000	0.083				
Perforin (% CD56^bright^ NK)	1.7	5.4	0.15–200	0.36				
CCL4 (% CD56^bright^ NK)	−2	0.13	0.012–1.5	0.098				
GMCSF (% CD56^bright^ NK)	−2	0.14	0.013–1.5	0.1				
IFNγ (% CD56^bright^ NK)	4.3	72	1.1–4700	0.044*				
CCL3 (% CD56^bright^ NK)	0.71	2	0.038–11	0.41				
TNFα (% CD56^bright^ NK)	1.9	6.9	0.35–140	0.21				

## Discussion

New therapies focussing on blocking inhibitory receptors not only of CD8 T cells but also of NK cells have shown clinical success^26^, supporting the notion that NK cells could have potentially important roles in future treatments. However, their implications in solid cancers is not entirely clear^32^. We analysed NK cells from a cohort of 29 late stage (III/IV) melanoma patients included in a vaccine clinical trial. All analysis was performed on samples from before vaccine treatment. In patients and healthy donors we found similar frequencies of circulating total NK cells as well as their subsets (CD56^dim^CD16^+^ and CD56^bright^), in line with previously published data^15,16^. CD56^dim^CD16^−^ NK cells were found reduced in viremic HIV patients^33^. We did not find significant differences between the frequencies of CD56^dim^CD16^−^ NK cells in healthy controls and melanoma patients. In the literature, NK cells are frequently associated with immune surveillance, often playing significant roles in tumour control^34^. Interestingly, we found that higher frequencies and numbers of circulating CD56^bright^ NK cells were associated with reduced overall and progression free patient survival, whereas frequencies or numbers of either total NK cells as well as CD56^dim^(CD16^+^ or CD16^−^) cells did not correlate with survival. A similar finding was previously reported by Tietze *et al*. who showed that low CD56^bright^ NK cell frequency at baseline is a good predictor of melanoma patient survival after treatment with Ipilimumab^27^.

Since CD56^bright^ NK cells adversely correlated with overall survival, we wondered if they possessed regulatory characteristics or if they had any deficiencies in their anti-tumoural potential. Previous studies in melanoma patients have shown decreased expression of NKp46 and NKG2D as well as an increased expression of NKp44 in total NK cells^15,16^. We did not observe differences in expression of NKp46 and NKG2D on CD56^bright^ NK cells. We found that CD56^bright^ NK cells expressed higher levels of CD38, CD11a and CD95, indicating that these cells were activated. We did not, however find evidence for increased apoptosis. CD38 is an ADP ribosyl-cyclase, which has been described to be involved in the production of adenosine, limiting CD4^+^ T cell proliferation as well as inducing regulatory T cells, mediated by CD56^bright^ cells^35^. Indeed, we found that CD38 expression levels on CD56^bright^ NK cells correlated with the frequencies of peripheral regulatory T cells from the same patients. Furthermore, CD11a was more strongly expressed on circulating CD56^bright^ NK cells in patients than healthy donors. It is one of the receptors (together with NKp46 and NKG2D) that could induce NK cell cytotoxicity directed towards antigen specific, activated T cells; especially CD4^+^ T cells seem to be sensitive to NK cell lysis in a mouse model of LCMV infection^36^. NK cell regulation of T cell function was shown during the priming phase^7^, and NK cell depletion during a persistent LCMV infection also led to a positive therapeutic effect^37^. Perforin was found to be the most important mediator of T cell and NK cell mediated killing^38^.

Apart from expression of surface markers, we also determined the functionality of healthy donor and patient-derived NK cells. We analysed the production of an array of cytokines and chemokines while making sure that ILCs did not interfere with our analysis. Previously, ILCs have not been gated out when determining cytokine production^15,16,31^. We found no differences in the amounts of CCL4 and IFNγ produced by circulating CD56^bright^ NK cells between patients and healthy donors. Unaffected production of IFNγ has been previously reported^39^. We found decreased production of TNFα and GMCSF by CD56^bright^ NK cells. Even though CD56^bright^ NK cells have a more activated phenotype in patients than healthy donors, they do not produce more cytokines. They are even partially impaired. GMCSF and TNFα are both pro-inflammatory cytokines. GMCSF can act in a paracrine manner and recruit neutrophils, monocytes and lymphocytes as well as enhance their functions. It plays an important role in priming of T cells as well as the development of a Th1 response^40^. In a phase II clinical trial, the administration of GMCSF to late stage melanoma patients in combination with Ipilimumab proved to be more efficient than Ipilimumab alone^41^. TNFα is known for its direct effects on cancer cells and for shaping the immune response^42^. It is also an essential cytokine for the process of antigen cross-presentation by DCs to CD8^+^ T cells^43^. In a mouse melanoma model, mice deficient for MIP-1α/β had increased tumour growth as well as a higher incidence of metastases, also associated with lower local production of IFNγ, TNFα and IL-6^44^.

Univariate Cox regression analysis of multiple clinical and immunological parameters identified three significant factors, namely the presence of distant metastases, the frequency of CD56^bright^ NK cells and IFNγ production by CD56^bright^ NK cells. IFNγ is usually seen as a positive mediator of the anti-tumour immune response. However, it has been shown that IFNγ produced by decidual NK cells has multiple immunoregulatory functions including inhibition of Th17 cells^45^, as well as induction of angiogenesis and vessel remodeling^46^. The interaction between decidual NK cells and monocytes leads to the induction of regulatory T cells as well as apoptosis of effector T cells, dependent of the IFNγ pathway^47^. The fact that distant metastases correlate with poor patient survival was expected as this is well known; patients without vs. with distant metastases are in stage III vs. IV, respectively; they have significantly different survival^48,49^. We did not observe a difference in CD56^bright^ NK cell frequencies depending on the presence of distant metastases. Both are thus independent prognostic factors for overall survival of melanoma patients.

In primary tumours, NK cells are only present in low numbers, preferentially in the peritumoral area^16^. In lymph node metastases, NK cells can be found at similar frequencies as in healthy donor tissue, making up less than 5% of CD45^+^ cells^23^. Few specific markers exist to distinguish NK cell subsets in the TME^16^. As a result, it is challenging to detect NK cell subsets in histologic material or based on computational approaches using transcriptome data^50^. Moreover, NK cells display a certain degree of plasticity^51^. Therefore our study was focused on circulating NK cells, specifically on CD56^bright^ and CD56^dim^ NK cells, their phenotype and functionality. Improved detection methods and studies are required to determine their roles and functions in the TME.

Our data suggest that CD56^bright^ NK cells may have a negative effect on the anti-tumour response by inhibiting T cell responses, via CD38, perforin, CD11a and IFNγ. On the other hand they produce less GMCSF and TNFα, cytokines important in establishing an anti-tumour response. However, due to the limited amount of patients included in this study, these should be considered as preliminary findings and be confirmed in larger patient cohorts, including early disease patients. The effects on the anti-tumour immune response of CD56^bright^ NK cells warrant also further mechanistic research. Our study provides evidence that the frequencies and absolute numbers of circulating CD56^bright^ NK could be potential biomarkers in melanoma patients.

## Methods

### Melanoma patients

Blood was obtained from melanoma patients included in a phase I clinical trial (ClinicalTrials.gov; Identifier: NCT00112229) upon written informed consent. Eligibility criteria and study design has been previously described^28^. The study was designed, approved and conducted according to relevant regulatory standards approved by the Ethics Commission for Clinical Research of the Faculty of Medicine and University of Lausanne (Lausanne, Switzerland), Swissmedic (Swiss Agency for Therapeutic Product) and the Protocol Review Committee of the Ludwig Institute for Cancer Research (New York). The study was performed at the Centre Hospitalier Universitaire Vaudois (CHUV) in Lausanne. All 29 stage III/IV melanoma patients were included in the present study. Clinical details of all patients are assembled in Supplementary Table 1. Only samples from before the trial treatment were used in this study. Control PBMC from healthy donors where isolated from blood donations obtained from the Blood Transfusion center. The cells were isolated by density gradient centrifugation using Lymphoprep.

### Human cell preparation and flow cytometry

PBMCs were isolated from whole blood cells by Lymphoprep (Axis-Shield) centrifugation gradient and cryopreserved in liquid nitrogen. Frozen PBMCs were thawed in a water bath at 37 °C. They were stained immediately after thawing, first with antibodies directed against surface markers. The following antibodies were used for the NK cell phenotyping: anti-CD11a (BD Biosciences Cat# 347983, RRID:AB_400366), anti-PD-1 (BD Biosciences Cat# 557946, RRID:AB_647199), anti-CD56 (BioLegend Cat# 318322, RRID:AB_893389), anti-CD16 (Beckman Coulter Cat# A33098, RRID: AB_2728092), anti-NKp46 (BD Biosciences Cat#, RRID:AB_10894195), anti-NKG2D (BioLegend Cat# 320808, RRID:AB_492962), anti-CD8 (BioLegend Cat# 300920, RRID:AB_528885), anti-CD3 (Beckman Coulter Cat# A93687, RRID: AB_2728095), anti-CD127 (BioLegend Cat# 351310, RRID:AB_10960140), anti-CD14 (Beckman Coulter Cat# B01175, RRID: AB_2728099), anti-CD19 (Beckman Coulter Cat# A96418, RRID: AB_2728101), anti-CD123 (BD Biosciences Cat# 563072, RRID: AB_2728102), anti-CD141 (BD Biosciences Cat# 563298, RRID: AB_2728103), anti-KLRG1 (A488, clone 13F12F2, provided by H. Pircher), anti-CD158b (Beckman Coulter Cat# IM2278U, RRID: AB_2728104), anti-NKG2A (Miltenyi Biotech Cat# 130-105-647, RRID:AB_2655388), anti-CD57 (BD Biosciences Cat# 333169, RRID: AB_2728105), anti-CD38 (Thermo Fisher Scientific Cat# 25-0389-42, RRID:AB_1724057), anti-CD95 (BioLegend Cat# 305612, RRID:AB_314550). Secondly, a live/dead staining (LIVE/DEAD™ Fixable Near-IR Dead Cell Thermo Fisher Scientific Cat# L-34975) was performed. Cells were fixed at room temperature (RT) for 30 minutes (1% formaldehyde buffer). Cells were washed and stained with anti–CTLA-4 (BD Biosciences Cat# 555855, RRID: AB_398615), in FACS buffer with 0.1% saponin for 30 minutes at RT. The following antibodies were used for the NK cell functionality panel: anti-CD56 (BioLegend Cat# 318328, RRID:AB_11218798), anti-CD16 (BioLegend Cat# 302026, RRID:AB_2278418), anti-CD3 (Thermo Fisher Scientific Cat# 47-0036-42, RRID:AB_10717514), anti-CD137 (BioLegend Cat# 309826, RRID:AB_2566260), anti-CD69 (BioLegend Cat# 310926, RRID:AB_2074956), anti-NKp44 (BioLegend Cat# 325114, RRID:AB_2616752), anti-CD127 (BD Biosciences Cat# 563086, RRID: AB_2728655), anti-CD14 (Beckman Coulter Cat# B01175, RRID: AB_2728099), anti-CD19 (Beckman Coulter Cat# A96418, RRID: AB_2728101). Then a live/dead staining (LIVE/DEAD™ Fixable Aqua Dead Cell Stain Thermo Fisher Scientific Cat# L34965) was performed. Depending on the sample/panel, PBMCs were washed with Annexin V binding buffer (BD Biosciences) and stained for Annexin V (BD Biosciences Cat# 556419, RRID: AB_2665412). Cells were fixed at RT during 30 minutes (FoxP3 intracellular staining kit, eBioscience). Cells were washed and stained with; anti-TNFβ (Thermo Fisher Scientific Cat# BMS105FI, RRID:AB_10598519), anti-IFNγ (BioLegend Cat# 506804, RRID:AB_315454), anti-GMCSF (BioLegend Cat# 502310, RRID:AB_11150231), anti-IL-22 (R and D Systems Cat# IC7821P, RRID:AB_495011), anti-CCL4 (Thermo Fisher Scientific Cat# 46-7540-42, RRID:AB_2573845), anti-IL-13 (BD Biosciences Cat# 561162, RRID:AB_10642586), anti-perforin (BioLegend Cat# 308104, RRID:AB_314702), anti-IL-4 (BioLegend Cat# 500810, RRID:AB_315129), anti-granzyme B (Thermo Fisher Scientific Cat# GRB17, RRID:AB_2536540), anti-TNFα (BioLegend Cat# 502926, RRID:AB_2204081), anti-CCL3 (Miltenyi Biotech Cat# 130-103-631, RRID:AB_2651378) and anti-IL-5 (BD Biosciences Cat# 554396, RRID:AB_398548) in FoxP3 intracellular staining kit permeabilisation buffer (eBioscience) for 30 minutes at RT. CD4 regulatory T cells in patients PBMCs were identified *ex vivo* by staining with the following antibodies at the surface: anti-CD3 PerCP (BD Biosciences Cat# 345766, RRID:AB_2783791), anti-CD4 PE-Cy7 (Biosciences Cat# 348809, RRID: AB_2783789), anti-CD25 PE (BD Biosciences Cat# 341011, RRID: AB_2783790), anti-CD127 Pacific Blue (eBioscience Cat# 57-1278-73, AB_657602). Then a live/dead staining (LIVE/DEAD™ Fixable Aqua Dead Cell Stain Thermo Fisher Scientific Cat# L34965) was performed. The FoxP3 staining were performed after Fixation and Permeabilization using the FoxP3 intracellular staining kit permeabilisation buffer (eBioscience) using anti-FoxP3-FITC (eBioscience Cat# 11-5773-82, RRID: AB_11076963). Melan-A specific T cells were identified using tetramers as previously described^28^. In short: enriched CD8^+^ T cells were incubated with phycoerythrin-labeled HLA-A*0201/Melan-A/MART-1 A27L peptide26-35 (ELAGIGILTV) tetramers (1 µg ml^−1^, 60 min, 4 °C) and then with antibodies (30 min, 4 °C).

Data were acquired on a Gallios (Beckman Coulter) and LSR-II^TM^ Flow Cytometer (BD Bioscience) and analysed using FlowJo 10.4.2 (TreeStar).

### *In vitro* stimulation

PBMCs were isolated from whole blood and cryopreserved in liquid nitrogen. Frozen PBMCs were thawed in a water bath at 37 °C. Cells were kept at 37 °C overnight in 20U/ml human recombinant IL-2 (Proleukin) and RPMI (Gibco) with 10% FCS (Gibco). Cells were stimulated for 4 hours with 1 µg/ml Phorbol 12-Myristate 13-Acetate (PMA) (Sigma Aldrich) and 0.25 µg/ml Ionomycin, (Sigma Aldrich). 10 µg/ml of Brefeldin A (Sigma Aldrich) and 2 nM Monensin (Sigma Aldrich) were added to all conditions, as well as to the control conditions at the start of the stimulation.

### Statistics and analysis

Significance of single comparisons was assessed using the Mann–Whitney test, multiple comparisons were analysed using a two-way ANOVA or with a Kruskal-Wallis test, using the GraphPad Prism 8 software. Overall survival (OS) was defined as the time between enrolment in the phase I clinical trial and latest follow-up or death. Progression-free survival (PFS) was defined as the time between clinical trial enrolment and relapse or progression of disease, based on whether the patient had no evidence of disease or evidence of disease at study entry, respectively. Apart from the clinical parameters, our data did not follow a normal distribution but a log distribution and was therefore log-transformed before statistical testing. The significance of Kaplan-Meier survival analysis was assessed by the Log-rank test (Prism 8). Cox proportional hazards model was used to test association with survival. Predictors were selected for multivariate regression based on results from univariate analyses, inclusion was based on statistical significance. Survival analysis was performed using the survival R package^52^.

## Supplementary information

Supplementary information

## Data Availability

All data generated or analysed during this study are included in this published article (and its Supplementary Information Files).
