# Supplementary material for: Circulating CD56bright NK cells inversely correlate with survival of melanoma patients
Source: Sci Rep. 2019 Mar 14;9:4487. doi: 10.1038/s41598-019-40933-8 (PMC6418246; doi:10.1038/s41598-019-40933-8)
Supplement: Supplementary file 1 — Supplementary information [file 41598_2019_40933_MOESM1_ESM.docx]

Supplementary data of the manuscript

Circulating CD56^bright^ NK cells inversely correlate with survival of melanoma patients

Kaat de Jonge^1^, Anna Ebering^1^, Sina Nassiri^1,3^, Hélène Maby-El Hajjami^1^, Hajer Ouertatani-Sakouhi^1^, Petra Baumgaertner^1^ and Daniel E. Speiser^1,2*^

^1^ Dept. of Fundamental Oncology, University of Lausanne, Epalinges, Switzerland

^2^ Dept. of Oncology, University Hospital Center (CHUV), Lausanne, Switzerland

^3^ Swiss Institute of Bioinformatics (SIB), Bâtiment Génopode, Lausanne, Switzerland

*** Corresponding author:** Daniel E. Speiser, Clinical Tumor Biology & Immunotherapy Group, Department of Oncology and Ludwig Cancer Research, University of Lausanne, Chemin des Boveresses 155, CH-1066 Epalinges, Switzerland, Phone: +41 21 314 01 82, Fax: +41 21 692 59 95, E-mail: doc@dspeiser.ch

**Table of contents:**

Supplementary Figure 1 2

Supplementary Figure 2 4

Supplementary Figure 3 5

Supplementary Figure 4 6

Supplementary Table 1 7


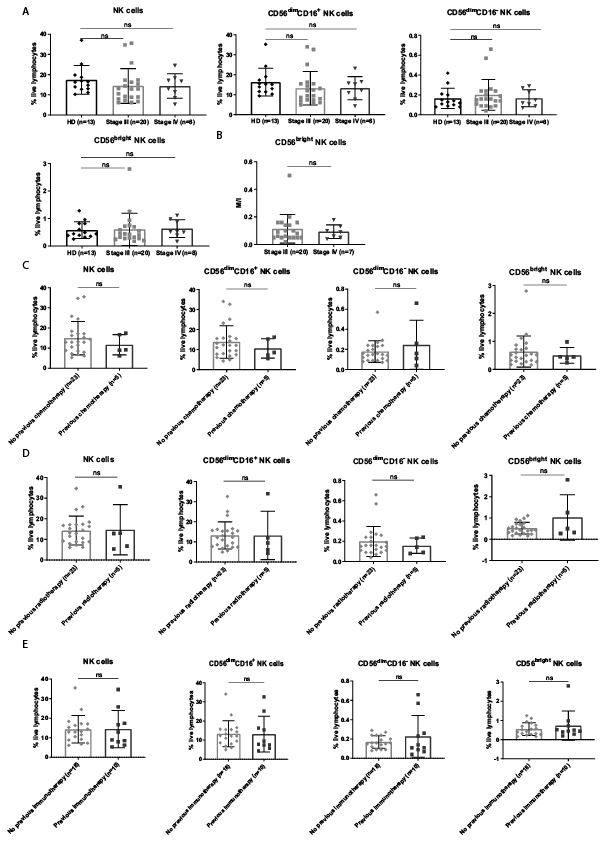


Supplementary Figure 1: Distribution of total NK cells, CD56^bright^, CD56^dim^CD16^+^ and CD56^dim^CD16^-^ NK cells in different clinical conditions. A. Distribution of NK cell frequencies and their subsets (%) from patients with late stage (III/IV) melanoma and healthy donors. B. Distribution of the absolute numbers of CD56^bright^ NK cells in stage III and stage IV melanoma patients. C,D,E. Distribution of total NK cells, CD56^bright^, CD56^dim^CD16^+^ and CD56^dim^CD16^-^ NK cells (%) in relation to previous chemotherapy (C), radiotherapy (D) or immunotherapy (E). ns not significant, * p<0.05, ** p<0.01, *** p<0.001, **** p<0.0001.


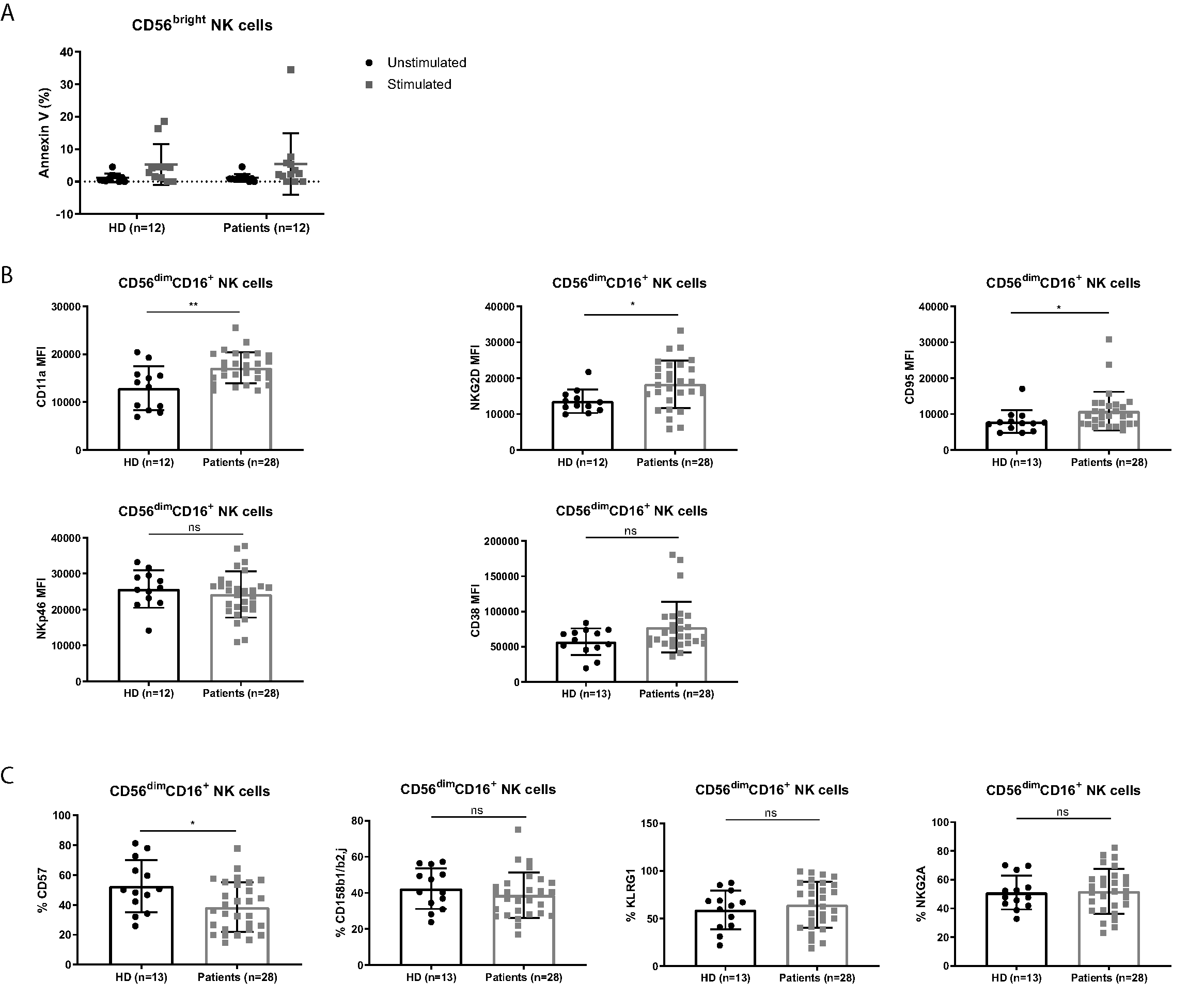


Supplementary Figure 2: Phenotypical characterization by flow cytometry of NK cells. A. Annexin V expression levels on CD56^bright^ NK cells in patients and healthy donors before and after activation (PMA/Ionomycin, 4 hours) B. Expression levels (MFI) of CD11a, NKG2D, CD95, NKp46 and CD38 on CD56^dim^CD16^+^ NK cells. C: Summary histograms of the expression (%) of CD57, CD158b1,b2,j, KLRG1 and NKG2A on CD56^dim^CD16^+^ NK cells. ns not significant, * p<0.05, ** p<0.01, *** p<0.001, **** p<0.0001.


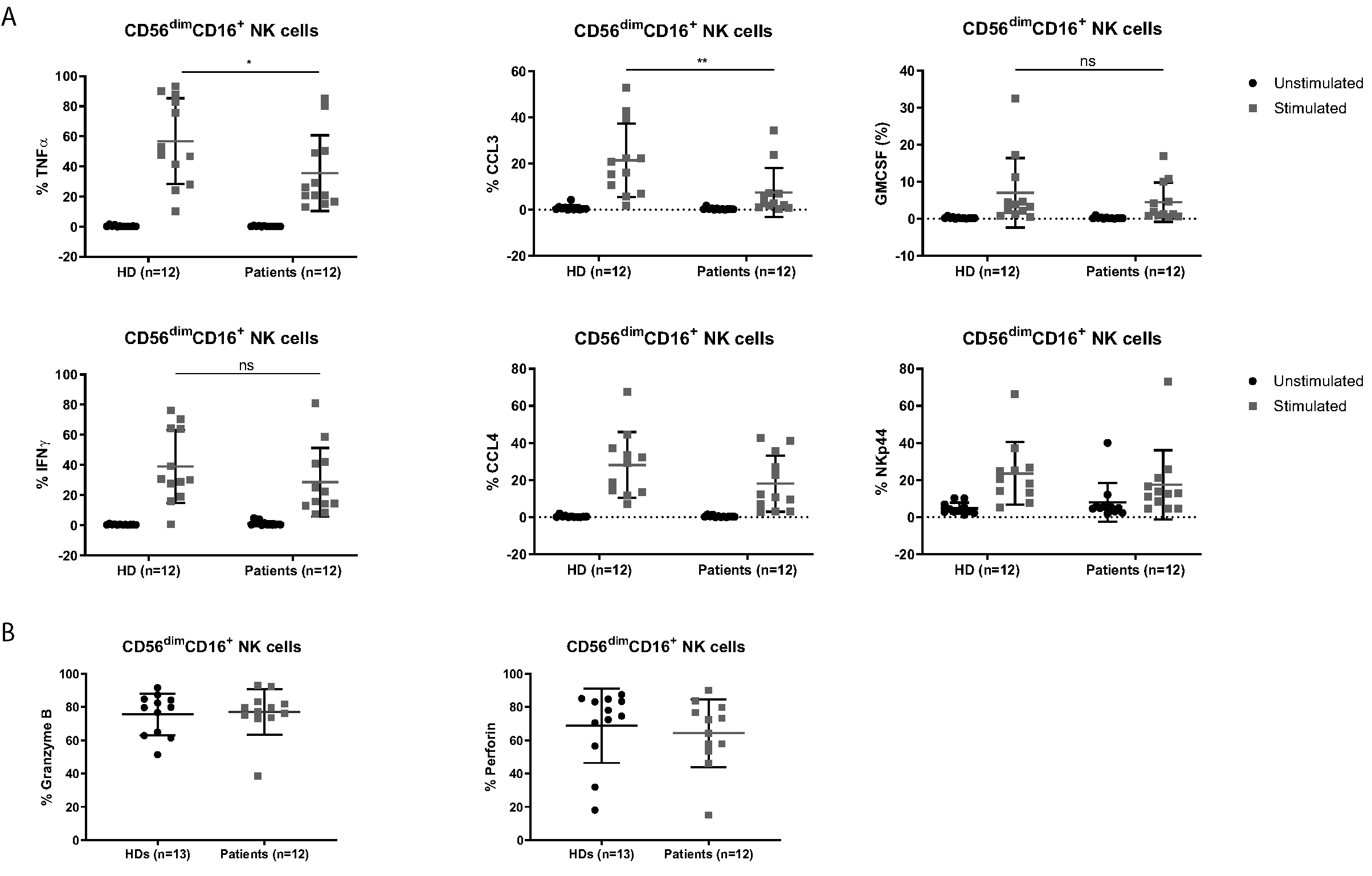


Supplementary Figure 3: Functional characterization of CD56^dim^CD16^+^ NK cells by flow cytometry. A. Histograms of TNFα, GMCSF, CCL3, CCL4 and IFNγ production (%) (CD56^dim^CD16^+^) as well as the expression level of NKp44 (%) in healthy donors and patients before and after stimulation (4h PMA/Ionomycin). B. Histograms of the expression levels of granzyme B and perforin by CD56^dim^CD16^+^ NK cells. ns not significant, * p<0.05, ** p<0.01, *** p<0.001, **** p<0.0001.


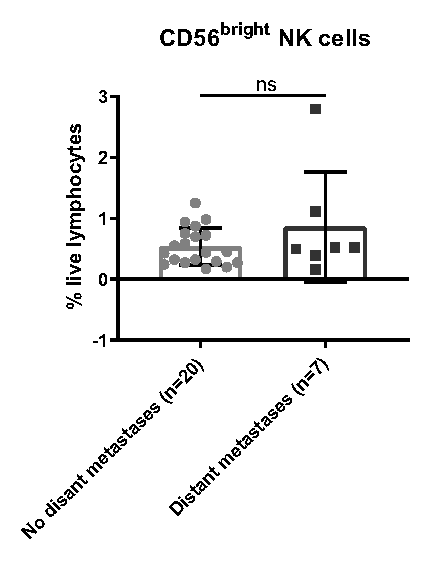


Supplementary Figure 4: CD56^bright^ NK cells (%) from melanoma patients with or without distant metastases. ns not significant, * p<0.05, ** p<0.01, *** p<0.001, **** p<0.0001.

**Supplementary Table 1: Clinical characteristics of the 29 late stage (III/IV) melanoma patients included in this study**

|  |  | Diagnosis | | | | | | Status at study entry | | | | | | Study outcome | | | |
| --- | --- | --- | --- | --- | --- | --- | --- | --- | --- | --- | --- | --- | --- | --- | --- | --- | --- |
| Patient | **Sex** | **Age** | **Melanoma type^‡^** | **TNM** | **Stage** | **Breslow** | **Clark** | **Age** | **TNM** | **Stage** | **Disease status^#^** | **Previous treatment^¤^** | **Study entry date** | **Death (1=yes/ 0=no)** | **OS^*^** | **Relapse (1=yes/ 0=no)** | **PFS^*^** |
| LAU 205 | M | 24 | SSM | pT2aN1bM0 | IIIB | 1.40 | IV | 33 | pT2aN2cM0 | IIIB | NED | IFNa adjuvant, Immuno-therapy (a) | 09.03.2005 | 1 | 50.6 | 1 | 25.2 |
| LAU 321 | M | 60 | SSM | pT3aN0M0 | IIA | 1.50 | III | 69 | pT3aN3M1b | IV | ED | Immuno-therapy (b), Chemo-immuno-therapy (c) | 30.06.2003 | 0 | 75.5 | 1 | 3.2 |
| LAU 371 | M | 29 | SSM | pT3aN1bM0 | IIIB | 2.38 | IV | 33 | pT3aN1bM1b | IV | NED | Immuno-therapy (d) | 28.07.2003 | 1 | 11.9 | 1 | 3.6 |
| LAU 392 | F | 29 | SSM | pT3aN0M0 | IIA | 2.50 | IV | 37 | pT3aN3M0 | IIIC | ED | Immuno-therapy (b) | 16.09.2004 | 1 | 11.8 | 1 | 2.5 |
| LAU 444 | F | 27 | NM | pT3aN0M0 | IIA | 1.90 | IV | 33 | pT3aN2cM1c | IV | ED | Radiotherapy, Immuno-therapy (e) | 15.09.2003 | 1 | 30.8 | 1 | 15.2 |
| LAU 618 | F | 69 | NM | pT4aN0M0 | IIB | 8.00 | V | 74 | pT4aN2cM0 | IIIB | ED | IFNa adjuvant, Chemo-immuno-therapy (c) | 26.05.2003 | 1 | 35.5 | 1 | 1.4 |
| LAU 627 | M | 49 | SSM | pT3bN1aM0 | IIIB | 2.23 | IV | 51 | pT3bN2bM1b | IV | ED | NA | 19.05.2003 | 1 | 15.2 | 1 | 3.4 |
| LAU 648 | M | 64 | UK | pT2aN0M0 | IB | 1.60 | IV | 70 | pT2aN3M0 | IIIC | NED | Radiotherapy, Immuno-therapy (e) | 25.10.2004 | 0 | 153.7 | 0 | 153.7 |
| LAU 660 | F | 22 | NM | pT2bN0M0 | IIA | 1.72 | IV | 25 | pT2bN0M1c | IV | NED | NA | 16.08.2004 | 1 | 14.8 | 1 | 2.1 |
| LAU 672 | M | 34 | SSM | pT1aN0M0 | IA | 0.70 | III | 38 | pT1aN3M0 | IIIC | ED | IFNa adjuvant, Immuno-therapy (f), Chemo-immuno-therapy (c) | 14.10.2003 | 1 | 45.5 | 1 | 1.9 |
| LAU 701 | F | 70 | UK | pT3bN0M0 | IIB | 2.50 | IV | 71 | pT3bN3M0 | IIIC | NED | Chemo-immuno-therapy (c) | 24.09.2003 | 0 | 140.6 | 1 | 2.9 |
| LAU 706 | F | 64 | UK | pTxN0M0 | UK | UK | UK | 67 | pTxN3M0 | IIIC | ED | Chemo-immuno-therapy (c), Immuno-therapy (a) | 08.11.2004 | 1 | 44.1 | 1 | 3.8 |
| LAU 818 | M | 55 | UK | pT3aN0M0 | IIA | 2.44 | III | 58 | pT3aN2bM0 | IIIB | NED | NA | 09.05.2003 | 0 | 61.2 | 1 | 7.3 |
| LAU 936 | F | 52 | SSM | pT3aN0M0 | IIA | 2.70 | IV | 54 | pT3aN1bM0 | IIIB | NED | Radiotherapy | 08.03.2006 | 1 | 15.1 | 1 | 1.5 |
| LAU 944 | F | 20 | OM | pT2aN0M0 | IB | 6.80 | UK | 28 | pT2aN1bM0 | IIIB | NED | Radiotherapy | 26.01.2004 | 0 | 168.0 | 1 | 23.8 |
| LAU 972 | F | 60 | NM | pT2bN1aM0 | IIIB | 1.60 | III | 60 | pT2bN1aM0 | IIIB | NED | NA | 02.09.2004 | 0 | 129.3 | 0 | 129.3 |
| LAU 975 | M | 51 | NM | pT4aN1bM0 | IIIB | 12.00 | IV | 52 | pT4N1bM0 | IIIB | NED | NA | 11.04.2005 | 1 | 7.5 | 1 | 4.2 |
| LAU 1013 | M | 55 | SSM | pT3bN3M0 | IIIC | 3.00 | IV | 56 | pT3bN3M0 | IIIC | NED | NA | 25.04.2005 | 1 | 25.1 | 1 | 8.8 |
| LAU 1015 | M | 75 | SSM | pT2aN0M1a | IV | 1.20 | III | 75 | pT2aN0M1a | IV | NED | NA | 03.03.2005 | 1 | 50.7 | 1 | 8.9 |
| LAU 1017 | F | 28 | NM | pT3bN2bM0 | IIIC | 3.80 | IV | 28 | pT3bN2bM0 | IIIC | NED | NA | 25.04.2005 | 1 | 22.0 | 1 | 1.4 |
| LAU 1022 | M | 69 | NM | pT2bN2bM0 | IIIB | 1.49 | IV | 69 | pT2bN2bM0 | IIIB | NED | NA | 11.07.2005 | 1 | 19.8 | 1 | 8.8 |
| LAU 1034 | M | 47 | SSM | pT2aN2bM0 | IIIB | 1.35 | III-IV | 47 | pT2aN2bM0 | IIIB | NED | NA | 15.08.2005 | 0 | 117.2 | 1 | 51.8 |
| LAU 1090 | M | 68 | NM | pT3aN2bM0 | IIIB | 3.10 | IV | 69 | pT3aN2bM0 | IIIB | ED | NA | 20.02.2006 | 1 | 21.0 | 1 | 3.8 |
| LAU 1106 | M | 36 | SSM | pT2aN1aM0 | IIIA | 1.35 | IV | 36 | pT2aN1aM0 | IIIA | NED | NA | 21.03.2006 | 0 | 106.8 | 0 | 106.8 |
| LAU 1129 | M | 52 | SSM | pT3N0M0 | II | 2.50 | IV | 66 | pT3N3M0 | IIIC | NED | Chemotherapy (g) | 29.06.2006 | 1 | 17.0 | 1 | 9.4 |
| LAU 1144 | M | 68 | NeM | pT3aN0M0 | IIA | 0.60 | IV | 72 | pT3aN0M1b | IV | NED | NA | 19.09.2006 | 1 | 29.4 | 1 | 8.9 |
| LAU 1164 | M | 51 | UK | pTxNxM1a | IV | UK | UK | 52 | pTxNxM1a | IV | NED | NA | 16.10.2006 | 0 | 56.6 | 0 | 56.6 |
| LAU 1189 | F | 68 | ALM | pT3bN2M0 | IIIB | 4.00 | IV | 68 | pT3bN2M0 | IIIB | NED | NA | 05.06.2007 | 0 | 38.9 | 1 | 10.9 |
| LAU 1264 | M | 46 | SSM | pT3bN0M0 | IIB | 4.00 | IV | 48 | pT3bN1bM0 | IIIC | NED | Radiotherapy | 11.10.2007 | 0 | 90.8 | 1 | 15.8 |

**^‡^** The melanoma type is shown: ALM: acral lentiginous melanoma, NM: nodular melanoma, NeM: nevoid melanoma, OM: ocular melanoma, SSM: superficial spreading melanoma
UK: unknown.
**^#^** The disease status before the start of the vaccination trial is presented: NED: no evidence of disease; ED: evidence of disease.
**^¤^** Previous therapies are listed: All patients underwent surgery.
a: P40/ELA cancer vaccine study: Melan-A 26-35 (A27L) analog peptide (ELA)+ P40 adjuvant
b: LUDWIG 96-010 cancer vaccine study: Melan-A 26-35 (A27L) analog peptide (ELA)+ FluMa 58-66 peptide + low dose rhIL-2+ SB AS-2
c: Isolated limb perfusion with Melphalan+IFNγ+TNFα
d: LUDWIG 98-009 cancer vaccine study: Melan-A 26-35 (A27L) analog peptide (ELA)+FluMa 58-66 peptide + SB AS-2
e: LUDWIG 96-010 cancer vaccine study: Melan-A 26-35 (A27L) analog peptide (ELA)+ FluMa 58-66 peptide + low dose rhIL-2+ Montanide ISA-51
f: LUDWIG 96-010 cancer vaccine study: Melan-A 26-35 (A27L) analog peptide (ELA)+ FluMa 58-66 peptide + Montanide ISA-51
g: Cisplatine+ Dacarbazine+ Methotrexate
NA (not applicable) means no systemic treatment was administered before the start of the vaccination trial.
The study outcome with overall survival (OS) and progression-free survival (PFS) is displayed. **^*^** Interval from the start of vaccination protocol to event (in months)
